# Effects of the Tanaka Line on the genetic structure of *Bombax ceiba* (Malvaceae) in dry‐hot valley areas of southwest China

**DOI:** 10.1002/ece3.3888

**Published:** 2018-03-01

**Authors:** Miao‐Miao Ju, Yi Fu, Gui‐Fang Zhao, Cheng‐Zhong He, Zhong‐Hu Li, Bin Tian

**Affiliations:** ^1^ Key Laboratory for Forest Resources Conservation and Utilisation in the Southwest Mountains of China Ministry of Education Southwest Forestry University Kunming China; ^2^ Key Laboratory of Biodiversity Conservation in Southwest China State Forestry Administration Southwest Forestry University Kunming China; ^3^ Key Laboratory of Resource Biology and Biotechnology in Western China College of Life Sciences Northwest University Xi'an China; ^4^ Key Laboratory of Biodiversity and Biogeography Kunming Institute of Botany Chinese Academy of Sciences Kunming China

**Keywords:** *Bombax ceiba*, genetic diversity, population structure, simple sequence repeats, Tanaka Line

## Abstract

Southwest China is an important biodiversity hotspot. The interactions among the complex topography, climate change, and ecological factors in the dry‐hot valley areas in southwest China may have profoundly affected the genetic structure of plant species in this region. In this study, we determined the effects of the Tanaka Line on genetic variation in the wild *Bombax ceiba* tree in southwest China. We sampled 224 individuals from 17 populations throughout the dry‐hot valley regions. Six polymorphic expressed sequence tag–simple sequence repeat primers were employed to sequence the PCR products using the first‐generation Sanger technique. The analysis based on population genetics suggested that *B. ceiba* exhibited a high level of gene diversity (*H*_E_: 0.2377–0.4775; *I*: 0.3997–0.7848). The 17 populations were divided into two groups by cluster analysis, which corresponded to geographic characters on each side of the Tanaka Line. In addition, a Mantel test indicated that the phylogeographic structure among the populations could be fitted to the isolation‐by‐distance model (*r* ^2^ = .2553, *p *< .001). A barrier test indicated that there were obstacles among populations and between the two groups due to complex terrain isolation and geographic heterogeneity. We inferred that the Tanaka Line might have promoted the intraspecific phylogeographic subdivision and divergence of *B. ceiba*. These results provide new insights into the effects of the Tanaka Line on genetic isolation and population differentiation of plant species in southwest China.

## 1 INTRODUCTION

Geographic isolation due to the uplift of mountain chains and climatic fluctuations associated with glacial oscillations can cause great variations in both the morphology and geographic distribution of many species (Liu et al., 2013). Genetic differentiation in local species may be the result of mutation or genetic drift, while gene flow may adapt to the native conditions to either restrict evolution or accelerate evolution by spreading new genes (Montgomery, 1987). The adaptation of species to a specific geographic habitat is partly determined by a series of historical events. In most cases, a species will extend the range of its habitat until barriers prevent dispersal, where the obstacles are mostly large and conspicuous, such as high mountains, wide oceans, deserts, or other geographic features, which form a relatively isolated network region (He & Jiang, 2014). Studies have also shown that species distribution patterns and structures are also affected by many external ecological factors, including climate, predators, and competitors (Montgomery, 1987).

Southwest China is one of the most important biodiversity hotspots, and it is characterized by extremely complex geographically isolated habitats (He & Jiang, 2014). The altitudes in this region differ greatly where they range from 300 m in Nujiang valley to Mount Gongga at more than 7,556 m above sea level (Zhao & Yang, 1997). Most of these parallel mountain chains are oriented in a north–south direction, and they are divided by very deep river canyons. The highly complex terrains may provide a relatively stable model of ecologically diverse habitats and glacial refugia because the vegetation and habitats only shifted vertically by a few hundred meters during the Pleistocene climate fluctuation (He & Jiang, 2014). Thus, this particular geographic environment is a natural cradle that maintains species richness. A well‐known biogeographic boundary exists in southwest China known as the Tanaka Line (Tanaka, 1954; Zhu & Yan, 2002). The Tanaka Line is considered to be a straight line between approximately 28°N, 98°E and 18°45′N, and 108°E, which divides the two floristic subkingdoms of East Asia, with the Sino‐Japanese to the east and the Sino‐Himalayan to the west (Li & Li, 1997). The genetic diversity and population subdivisions are markedly different on either side of the Tanaka Line (Fan et al., 2013; Tian et al., 2015), which makes this an ideal region to study the effects of different factors on species diversification and evolution.

*Bombax ceiba* Linn. (Malvaceae), known as the red silk cotton tree, is a tall, drought‐tolerant, and arbor tree species with a wide distribution on both sides of the Tanaka Line (Chaudhary & Khadabadi, 2012). Natural populations of this tree species are widespread in South‐East Asian countries at altitudes below 1,400–1,700 m (Li, 1984). In China, *B. ceiba* occurs naturally in subtropical regions, such as the dry‐hot valleys of Yunnan and adjacent provinces (Jin, Yang, & Tao, 1995). The specific habitat range of wild *B. ceiba* provides an opportunity to verify whether the Tanaka Line has acted as a geological or climatic barrier to affect population structure formation. In our previous study (Tian et al., 2015), we analyzed phylogeographic patterns based on three chloroplast DNA regions (*psb*B‐*psb*F, *trn*L‐*rpl*32, and *psb*I‐*psb*K) in 17 natural *B. ceiba* populations (201 individuals), where the results showed that the main reasons for differences in the genetic structure of *B. ceiba* either side of the Tanaka Line are historical climate change and complex topographical conditions (Tian et al., 2015). However, it is not clear whether there is an intraspecific divergence pattern in this species where apparent gene flow occurs across the Tanaka Line.

Genetic diversity determines population diversity and the genetic variation among populations or species (Meng et al., 2015; Zhang, Chen, Zhang, Chen, & Fang, 2011). The long‐term survival of wild species requires a rich gene pool with sufficient genetic diversity to adapt to continual environmental changes, thereby increasing the likelihood of survival or recovery (Cruz et al., 2012). In the study, we aimed to determine the population structure and genetic variation in wild *B. ceiba* resources in order to facilitate conservation strategies. We used six pairs of expressed sequence tag–simple sequence repeat (EST‐SSR) primers to determine the population structure and diversity of wild *B. cieba* populations on both sides of the Tanaka Line. Moreover, the PCR products were subjected to Sanger sequencing to make the results more accurate and reliable (Hutchison, 2007).

## 2 MATERIALS AND METHODS

### 2.1 Plant materials

**Figure 1:**
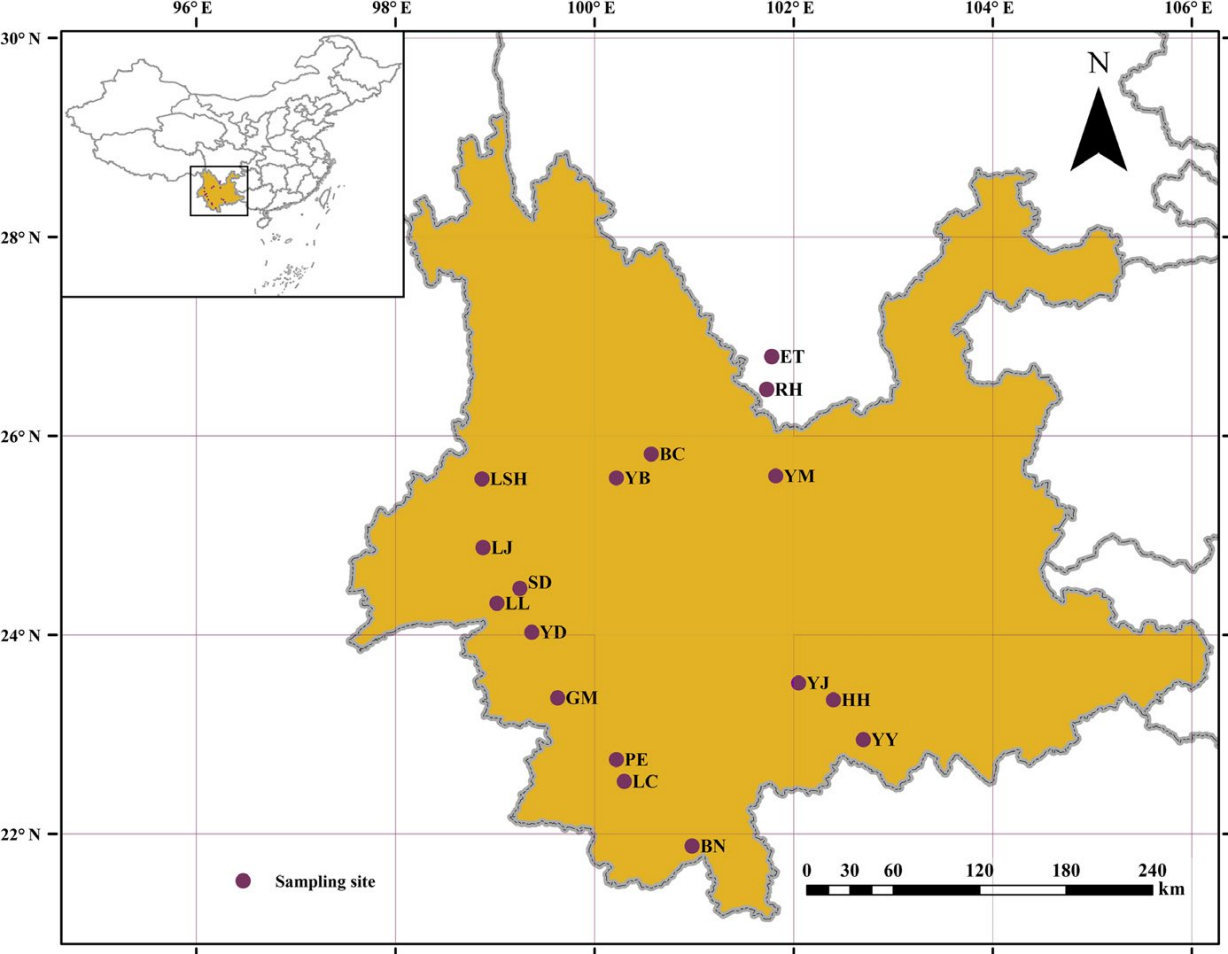
Geographic distribution of *B. ceiba*. Each dot represents a natural population sampled in this study

We collected 224 samples from 17 populations of *B. ceiba* in the dry‐hot valleys of southwest China (Figure 1). The fresh leaf sample was placed onto silica gel and dried immediately. The latitude and longitude were recorded for each sampled population using GPS system (Garmin, Taiwan), and the locations are listed in Appendix 1. Voucher specimens were preserved and archived in the herbarium of Southwest Forestry University, China.

### 2.2 DNA extraction, PCR amplification, and sequencing

Total genomic DNA was extracted from leaf tissues using DNA Extraction Kits (TIANGEN, Beijing, China) according to the manufacturer's protocol. The concentration and quality of the DNA were detected using a spectrophotometer. Six highly variable pairs of EST‐SSR primers (Appendix 2) were used to detected polymorphisms in *B. ceiba*. PCR amplification was performed according to the procedure described previously by Ju, Ma, Xin, Zhou, and Tian (2015). All of the high‐quality PCR products were sequenced using the amplified forward and reverse primers with an ABI 3730xl Sequence Analyzer (Life Technologies, Carlsbad, CA, USA).

### 2.3 Data analysis

The sequences obtained were aligned using MUSCLE (Edgar, 2004) and revised manually in MEGA 7 (Tamura et al., 2011). Hardy–Weinberg equilibrium and linkage disequilibrium were assessed for each population and microsatellite locus pair with PopGen version 32 (Yeh, Yang, & Boyle, 1999). Neutral microsatellite loci were used for the population genetic analyses. Genetic diversity parameters comprising the allele size (*A*), effective number of alleles (*N*_e_), observed heterozygosity (*H*_o_), expected heterozygosity (*H*_e_), and polymorphism information content (PIC) were calculated for each locus using GenAlEx version 6.501 (Peakall & Smouse, 2012) and PIC_CALC version 0.6. Correlation analyses of the genetic similarity and geographic distances among the 17 populations were calculated using PopGen version 32 and based on a Mantel test (Mantel, 1967) with 999 matrix randomizations using GenAlEx version 6.501. According to the genetic distance matrix calculated among the 224 samples with GenAlEx version 6.501, the similarity matrix was subjected to cluster analysis using the UPGMA algorithm with NTSYS‐PC version 2.0 and a dendrogram was generated (Rohlf, 2000). Interpopulation and intrapopulation genetic differentiation were partitioned by analysis of molecular variance (AMOVA) using ARLEQUIN version 3.5.2.1 (Excoffier & Lischer, 2010) with 1,000 random permutation tests. The population genetic structure was determined with the Bayesian clustering approach implemented in STRUCTURE version 2.3.1 (Evanno, Regnaut, & Goudet, 2005). An admixture ancestry model was applied, and 10 independent runs were conducted for each *K* (1–9) with 50,000 burn‐in and 100,000 Markov Chain Monte Carlo iterations. A suitable number of clusters (*K*) were selected as the largest rate of change in the log probability of data between successive *K* values (Pritchard, Stephens, & Donnelly, 2000), as implemented in STRUCTURE HARVESTER (available online at: http://taylor0.biology.ucla.edu/structureHarvester/). Admixture proportions obtained from replicate simulations at the optimal *K* were averaged using CLUMPP version 1.1.2 (Jakobsson & Rosenberg, 2007). We then employed MIGRATE‐N v3.6 (Beerli, 2006) to explore the direction of historical gene flow among the 17 populations based on the Bayesian clustering results. The geographic locations of genetic discontinuities among populations were determined with BARRIER version 2.2 (Manni, Guerard, & Heyer, 2004).

## 3 RESULTS

### 3.1 Genetic diversity

In total, 27 alleles were identified in the six SSR loci among the 224 individuals from 17 *B. cieba* populations. All of the loci conformed to Hardy–Weinberg equilibrium, and they were polymorphic among populations. The number of alleles (*A*) ranged from two to seven (Table 1), and the mean number of alleles was 4.5. *H*_o_ and *H*_e_ varied from 0.2217 to 0.4486 (mean value = 0.3620) and 0.2424 to 0.6085 (mean value = 0.4622), respectively (Table 1). The PIC value for each locus ranged from 0.2311 to 0.5534, with an average of 0.3874 (Table 1).

**Table 1:** Description of the six SSR primer combinations used for analyzing *B. ceiba*

Locus	Sample size	*A*	*N*_e_	*H*_o_	*H*_e_	PIC
BC1	408	5	1.9435	0.3382	0.4867	0.3974
BC5	442	7	1.3190	0.2217	0.2424	0.2311
BC9	428	4	2.5453	0.4486	0.6085	0.5534
BC10	440	6	2.1617	0.4455	0.5386	0.4396
BC11	410	2	1.8274	0.3610	0.4539	0.3503
BC12	420	3	1.7926	0.3571	0.4432	0.3524
Mean	425	4.5	1.9316	0.3620	0.4622	0.3874

*A*, number of alleles; *N*_e_, effective number of alleles; *H*_o_, observed heterozygosity; *H*_e_, expected heterozygosity; PIC, polymorphism information content.

The population genetic diversity results obtained at the population level are listed in Table 2, which show that there were clear differences in the numbers of polymorphic bands. The percentage of polymorphic loci per population varied from 66.67% to 100%. *N*ei's gene diversity (*H*_E_) and Shannon's index (*I*) ranged from 0.2377 to 0.4775 and 0.3997 to 0.7848, respectively (Table 2).

**Table 2:** Genetic diversity of natural populations of *B. ceiba*

Population	*N*	Number of polymorphic bands	PPB (%)	*H*_E_	*I*
BN	13	5	83.33	0.3467 ± 0.2410	0.5450 ± 0.3915
YJ	9	6	100	0.3940 ± 0.1554	0.6261 ± 0.2282
BC	17	6	100	0.4084 ± 0.1153	0.6577 ± 0.1936
LC	13	6	100	0.4178 ± 0.1171	0.6822 ± 0.1711
PE	12	6	100	0.4613 ± 0.1256	0.7668 ± 0.2632
YD	22	6	100	0.4775 ± 0.1214	0.7439 ± 0.1997
GM	19	6	100	0.4665 ± 0.1046	0.7362 ± 0.2309
HH	6	4	66.67	0.3244 ± 0.2513	0.4821 ± 0.3808
LJ	34	6	100	0.3333 ± 0.1418	0.5812 ± 0.2136
SD	6	6	100	0.4005 ± 0.1393	0.6233 ± 0.2339
LL	5	6	100	0.4658 ± 0.0509	0.7120 ± 0.1196
RH	9	6	100	0.2377 ± 0.1573	0.3997 ± 0.2081
LSH	14	6	100	0.3098 ± 0.1946	0.5550 ± 0.3248
YB	9	5	83.33	0.4537 ± 0.2923	0.7848 ± 0.5417
ET	13	5	83.33	0.3084 ± 0.1881	0.4810 ± 0.2840
YM	12	6	100	0.3592 ± 0.1559	0.6336 ± 0.2883
YY	11	5	83.33	0.3110 ± 0.2158	0.4802 ± 0.3235

*N*, sample size; PPB, percentage of polymorphic bands; *H*_E_, *N*ei's gene diversity; *I*, Shannon and Weaver's index.

### 3.2 Cluster analysis

**Figure 2:**
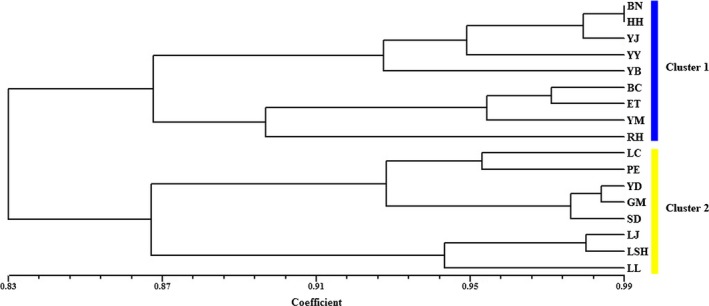
UPGMA dendrogram obtained for the *B. ceiba* populations based on Nei's genetic distance (Nei, 1972)

**Figure 3:**
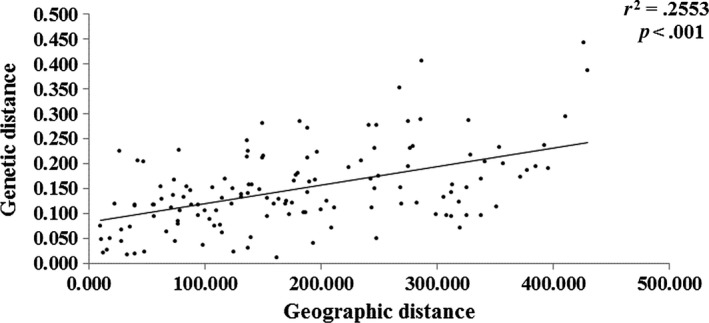
Mantel test between genetic distance and geographic distance among the 17 *B. ceiba* populations

Cluster analysis showed that all of the *B. ceiba* samples clustered into two groups (cluster 1 and cluster 2). Cluster 1 comprised the BN, HH, YJ, YY, YB, BC, ET, YM, and RH populations. The LC, PE, YD, GM, SD, LJ, LSH, and LL populations were grouped in cluster 2 (Figure 2). The UPGMA dendrogram suggested that the clustering was highly dependent on the geographic origins of populations (Figure 2). The populations northeast of the Tanaka Line belonged to cluster 1, whereas all of the populations southwest of the Tanaka Line belonged to cluster 2. Thus, the populations located on each side of Tanaka Line had relatively different genetic characters. The Mantel test results also indicated that there was a significant correlation between the genetic distances and geographic distances for all of the populations examined (*r* ^2^ = .2553, *p *< .001, 999 permutations) (Figure 3).

AMOVA detected genetic differentiation across the *B. ceiba* populations (Table 3). The results indicated a relatively low level of genetic differentiation among groups (2.69%), where 5.89% of the diversity was attributed to the population level and 91.42% was due to the genotypes within the populations. In addition, the population genetic differentiation within the northeast group was *F*_ST_ = 0.12069 and that among populations in the southwest group was *F*_ST_ = 0.06810.

**Table 3:** AMOVA test results for 224 *B. ceiba* individuals in the 17 natural populations

Regions	Source of variation	*df*	SSD	Variance component	Percentage variance (%)	Fixation Index
Whole	Among groups	1	5.817	0.01798	2.69	*F*_ST_: 0.08583
	Among populations	15	24.212	0.03930	5.89	*F*_SC_: 0.06052
	Within populations	431	262.949	0.61009	91.42	*F*_CT_: 0.02695
	Total	447	292.978	0.66737		
Northern region	Among populations	8	22.865	0.09829	12.07	*F*_ST_: 0.12069
	Within populations	189	135.347	0.71612	87.93	
Southern region	Among populations	7	14.458	0.04746	6.81	*F*_ST_: 0.06810
	Within populations	242	157.162	0.64943	93.19	

### 3.3 Population genetic structure

**Figure 4:**
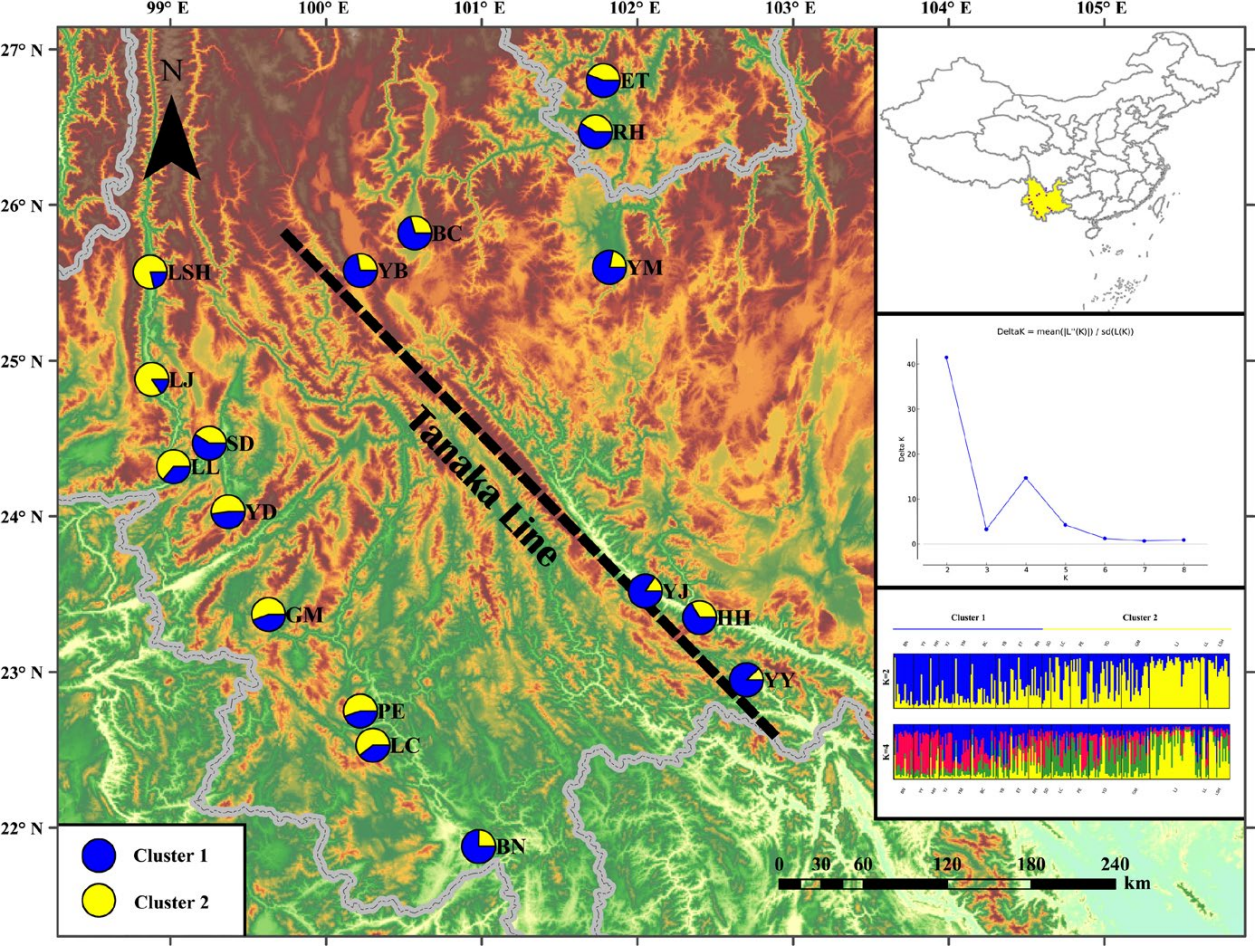
STRUCTURE clustering analysis results for *B. ceiba* populations based on their geographic distribution. Colors represent the population's probability of populations belonging to either of the two clusters, where blue represents cluster 1 and yellow represents cluster 2. The right‐hand figure shows the number of clusters (*K*) determined for the *B. ceiba* populations analyzed was the highest peak that was at *K* = 2. Each vertical bar in the histogram represents a population

According to the six neutral polymorphic markers employed in the population genetic structure analyses using *K* values ranging from 1 to 10, the STRUCTURE simulation obtained the highest peak at *K* = 2 (Figure 4). The two *B. ceiba* subpopulations at *K* = 2 were attributed to those in the southwest and northeast regions with respect to the Tanaka Line. The membership results inferred that the genetic structure of *B. ceiba* matched with the geographic distribution (Figure 4). The estimation of gene flow suggested that gene exchange existed among the populations (*N*m = 1.1792; Table 4).

**Table 4:** Genetic differentiation and gene flow in the 17 *B. ceiba* populations

Locus	*F*_IS_	*F*_IT_	*F*_ST_	*N*m
BC1	0.0465	0.2717	0.2362	0.8086
BC5	0.0331	0.0951	0.0641	3.6508
BC9	0.1296	0.2665	0.1573	1.3393
BC10	0.0660	0.1464	0.0861	2.6535
BC11	−0.1405	0.1553	0.2593	0.7140
BC12	0.0690	0.2683	0.2140	0.9180
Mean	0.0439	0.2112	0.1749	1.1792

We defined two clusters based on the STRUCTURE results in order to evaluate the direction of historical gene flow among the 17 populations. The gene pool in cluster 1 was mostly attributed to the northeast group and that in cluster 2 was attributed to the southwest group. We performed maximum likelihood analyses with MIGRATE‐N using 10 short chains (5,000 trees) and three long chains (50,000 trees), where 10,000 trees were discarded as a burn‐in. Interesting patterns of historical gene flow were determined between the two groups where these patterns were relatively symmetrical with slight differences (Table 5). However, all of the slightly asymmetrical patterns were related to a population migration direction from the northeast group to the southwest group (*m*_12_ > *m*_21_).

**Table 5:** MIGRATE analysis for *B. ceiba* using SSR data

Parameter	Percentiles								
	0.005	0.025	0.05	0.25	MLE	0.75	0.95	0.975	0.995
*m*_12_	0.9401	1.0096	1.0469	1.1677	1.2573	1.3521	1.4998	1.5540	1.6768
*m*_21_	0.8679	0.9453	0.9824	1.0986	1.1868	1.2821	1.4339	1.4868	1.5950

*m*_12_, migration rate from cluster 1 to cluster 2; *m*_21_, migration rate from cluster 2 to cluster 1.

**Figure 5:**
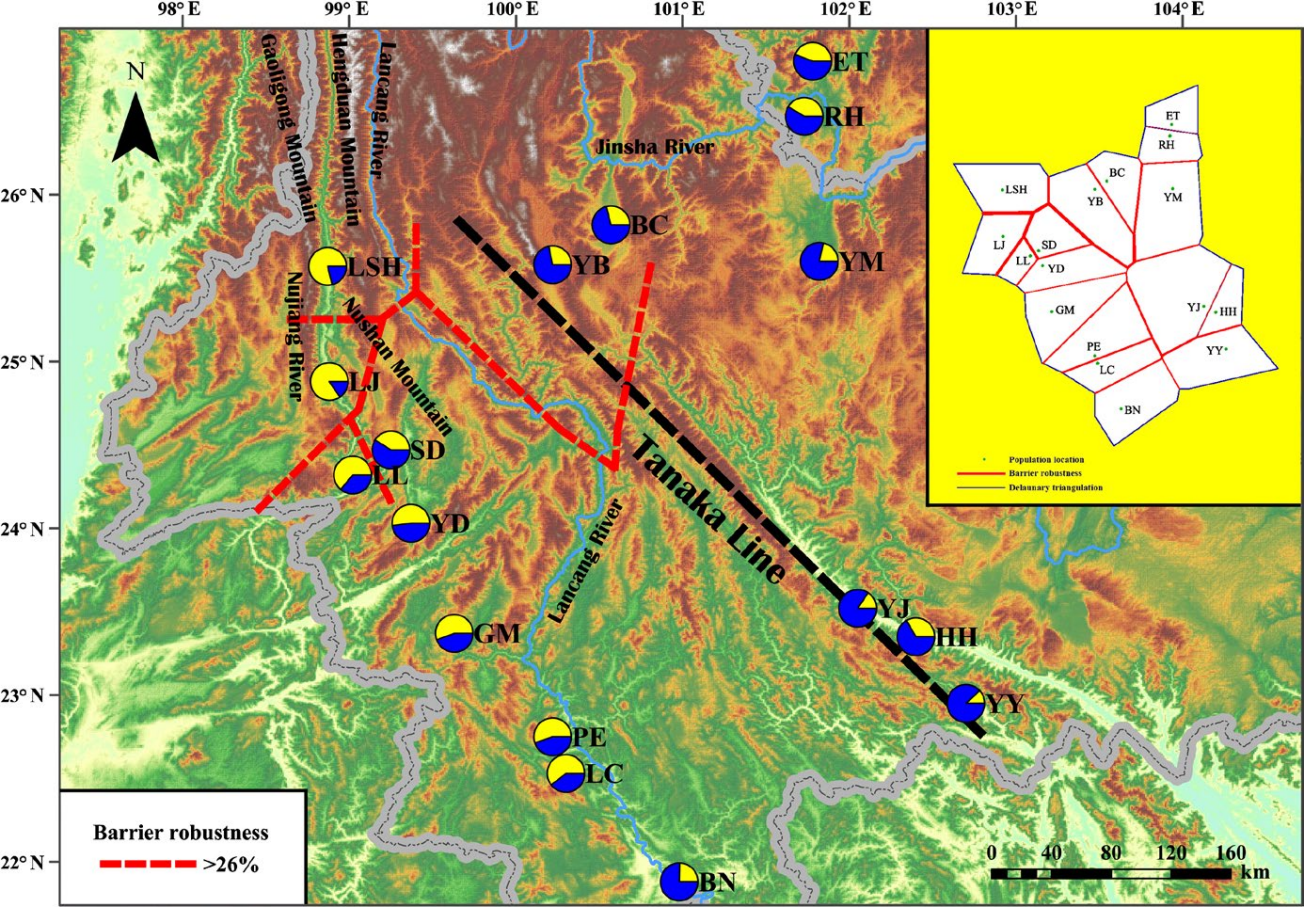
BARRIER analysis results showing the spatial separation. The BARRIER analysis results were based on microsatellite data (upper right)

BARRIER analysis suggested that the largest genetic breaks in many cases agreed with mountainous areas and rivers (Figure 5). Thus, the Gaoligong Mountains separated the LSH and LJ populations, Nujiang River isolated the LJ and LL populations, and the Nushan Mountains divided the LL and SD populations. These are the main barriers that have affected the dispersal of *B. ceiba*.

## 4 DISCUSSION

### 4.1 Genetic variation

Our analysis based on six microsatellite loci indicated that the PIC values ranged from 0.2311 to 0.5534, with a mean value of 0.3874 (Table 1). According to Botstein, White, Skolnick, and Davis (1980), all loci are considered to be reasonably informative. The mean values of *H*_o_ and *H*_e_ were 0.3620 and 0.4622, respectively (Table 1). Thus, the six SSR loci used for *B. ceiba* in this study exhibited high polymorphism.

Our analysis of the diversity among *B. ceiba* populations detected a high level of intrapopulation genetic diversity in *B. ceiba* (*H*_E_: 0.2377–0.4775; *I*: 0.3997–0.7848) (Table 2). In general, species with long history characteristic must have an adequate gene pool to provide sufficient diversity to survive and adapt to complex habitats (Booy, Hendriks, Smulders, Van Groenendael, & Vosman, 2000). Hence, it is important for plant species to retain as much genetic variation as possible to enhance its likelihood of recovery (Cruz et al., 2012). The natural *B. ceiba* populations had high genetic diversity in this study, possibly because this species is a perennial species with a high potential for outcrossing via entomophilous flowers (Aluri, Srungavarapu, & Kone, 2005). Previously, Nybom (2004) showed that perennial, outcrossing, and widely distributed species exhibit higher levels of genetic variability within populations. Furthermore, this high diversity may be the main factor that allowed *B. ceiba* to adapt to harsh environments and become the dominant species (Li, 1984) in these dry‐hot valleys through a long evolutionary process.

### 4.2 Population structure

Clustering analysis based on UPGMA and Bayesian methods suggested that the 17 natural *B. ceiba* populations could be divided into two genetically divergent clusters (Figures 2 and 4) located on either side of the Tanaka Line. This result is similar to that obtained based on chloroplast DNA data in a previous study by Tian et al. (2015). However, in the present study, we detected the gene flow among populations based on SSR markers. These two types of molecular markers differ in terms of genetic diversity and genetic differentiation, and they have been detected in various plants (Kurokawa, Kobayashi, & Ikeda, 2010; Zeinalabedini, Khayamnekoui, Grigorian, Gradziel, & Martinezgomez, 2010). Combining analyses based on nuclear and chloroplast markers can help to elucidate the evolutionary history of species with different inherited patterns (Mariana & Juan, 2016). Thus, in contrast to the chloroplast fragments, the DNA microsatellites could be used to determine contemporary pollen and seed dispersal (Wolfe, Li, & Sharp, 1987). Variation is influenced by the parental heredity and a high level of mutation rate, which reflects the current genetic structure and distribution of genetic variation (Mariana & Juan, 2016).

The *B. ceiba* flowers are red and cup‐shaped with rich nectar, and they could emit a mild fetid smell to attract a wide range of insects and animals, such as bees, birds, bats, and even monkeys (Aluri et al., 2005). While exploring the *B. ceiba* flowers, animals contact the stigma and stamens so the pollen can adhere to their head and body to facilitate dispersals. Some bees only collect nectar and move between conspecific trees nearby, thereby facilitating pollination (Aluri et al., 2005). This foraging behavior is considered to affect cross‐pollination, and it might weaken the genetic structure in the natural populations. In addition, Ashoke (1999) found that the highest number of pollen grains generated per flower by *B. ceiba* was about 8,863,000 and the maximum atmospheric incidence was 156/m^3^ at 10 hr.

In addition, the *F*_ST_ analysis showed that the proportion of genetic differentiation among populations accounted for about 0.1749 of the total genetic diversity (Table 4). According to Wright (1978), the differentiation among populations is relatively large (0.15–0.25). Clearly, a positive correlation between the genetic and geographic distances was detected among the populations (*r* ^2^ = .2553, *p *< .001) (Figure 3), and thus, topography may be one of the most important factors that have led to differentiation. Natural adaptation probably explains the first level of differentiation within the progenitor *B. ceiba* population, while habitat fragmentation may have been responsible for the second level of hierarchical variation. The isolation between populations is due to physical barriers in the form of complex terrain with mountains and rivers in southwest China. The genetic structure is expected to be congruent with the geographic arrangement of the mountains and river systems. The genetic distance was relatively large even with a close geographic distance, and genetic discontinuities between the two nearby territories were also identified by BARRIER (Figure 5).

BARRIER analysis based on microsatellite data showed that, in recent times, variations in the topography and climate have contributed to the high endemic biodiversity in southwest China (Myers, Mittermeier, Mittermeier, Da, & Kent, 2000). The extremely complex topography of this region provides ecologically diverse habitats in three dimensions. In addition, the river systems in southwest China are extremely complex, such as the Jinsha River, Lancang River, and the Nujiang River and its tributaries. River canyons have been shaped by tectonism to created uplifts on the Qinghai–Tibet Plateau, which may have existed prior to the rivers (Cheng, Liu, Gao, Tang, & Yue, 2001; Clark et al., 2004). These diverse and stable environments are highly favorable for maintaining species richness. In this study, the southwest and northeast regions contained two different genetic structures because of their terrain and large geographic barriers, such as the Hengduan Mountains and Lancang River. Between these two regions, the Tanaka Line may also be an important barrier that divides the *B. ceiba* natural populations into two parts. Indeed, previous studies have demonstrated that the Tanaka Line currently plays a key role in shaping plant dispersal and it is a habitat–heterogeneity boundary in southwest China (Tanaka, 1954; Zhu & Yan, 2002). The heterogeneous environmental conditions on the Tanaka Line have significantly affected the development and evolution of plant species, that is, a genetic diversity study of *Sophora davidii* found obvious differences in the population structure on both sides of the Tanaka–Kaiyong Line (Fan et al., 2013). Hence, the Tanaka Line may be responsible for maintaining the major southwest and northeast split in the *B. ceiba* populations associated with an ecological transition. This major form of isolation may hinder the gene exchange via birds but not pollen dispersal. Hence, this pattern may weaken the specific population structure of *B. ceiba* on either side of the Tanaka Line. Natural adaptation and physical barriers could explain the divergence among the two subpopulations. Overall, our findings support a hypothesis that the Tanaka Line has contributed to the intraspecific divergence pattern in this species, thereby facilitating the protection and exploitation of wild *B. ceiba* population resources.

## CONFLICT OF INTEREST

None declared.

## AUTHOR'S CONTRIBUTIONS

BT contributed to the conception of the study. BT and YF collected the materials. ZHL and MMJ contributed significantly to analysis and manuscript preparation. MMJ performed the data analyses and wrote the manuscript. CZH contributed the reagents/materials/analysis tools. BT, ZHL, and GFZ helped perform the analysis with constructive discussions. All authors contributed critically to the drafts and gave final approval for publication.

## APPENDIX 1 Locality information and numbers of *B. ceiba* sampled

### 1.1

**Table:**

Population code	Location	*N*	Geographic coordinates		
			Latitude (N)	Longitude (E)	Altitude (m)
BN	Xishuangbanna, Yunnan	13	21°53′	100°59′	570
YJ	Yuanjiang, Yunnan	9	23°31′	102°03′	850
BC	Binchuan, Yunnan	17	25°49′	100°34′	1,430
LC	Lancang, Yunnan	13	22°32′	100°18′	1,090
PE	Puer, Yunnan	12	22°45′	100°13′	1,340
YD	Yongde, Yunnan	22	24°02′	99°22′	1,110
GM	Gengma, Yunnan	19	23°22′	99°38′	890
HH	Honghe, Yunnan	6	23°21′	102°24′	520
LJ	Lujiang, Yunnan	34	24°53′	98°53′	660
SD	Shidian, Yunnan	6	24°28′	99°15′	1,100
LL	Longling, Yunnan	5	24°19′	99°01′	750
RH	Renhe, Sichuan	9	26°28′	101°44′	1,110
LSH	Lushui, Yunnan	14	25°34′	98°52′	1,060
YB	Yangbi, Yunnan	9	25°35′	100°13′	2,100
ET	Ertan, Sichuan	13	26°48′	101°47′	1,100
YM	Yuanmou, Yunnan	12	25°36′	101°49′	1,120
YY	Yuanyang, Yunnan	11	22°57′	102°42′	600

## APPENDIX 2 Characteristics of nuclear microsatellites used to analyze *B. ceiba*

### 2.1

**Table:**

Locus	Primer sequence (5′→3′)	Repeat motif	Allele size range (bp)	Ta (°C)	Fluorescent dye	BLAST to hit description [organism]
BC1	F: TACTCCGAAACTCACGCCTT	(CTT)_7_	270–273	59	6‐FAM	Nonintrinsic ABC protein 6, putative isoform 2 [*Theobroma cacao*]
	R: AAAGACGTATCGGTGAAGCG					
BC5	F: ACAAAAATGGCGTCTTCTGG	(CAG)_7_	128–134	60	6‐FAM	—
	R: GCAGGAGATCCATGGTGATT					
BC9	F: TTTGAAAGGGAGGGTGTTTG	(GACT)_6_	134–138	57	HEX	—
	R: GAGGAGGAAAAGTTATGTTTTGG					
BC10	F: ACCTCCTGCACAGACCATTC	(ACA)_8_	213–216	60	6‐FAM	—
	R: CATGGGGGAAAATTTTGTTG					
BC11	F: TGGGAGCTGAGATTTGATCC	(CAGC)_6_	316–320	60	NED	—
	R: CCCCACTGGATTGATTGATT					
BC12	F: TCCATCCAAATTCAGCAACA	(CAG)_8_	147–150	60	HEX	Auxin efflux facilitator isoform 6 [*Theobroma cacao*]
	R: GGTTTGCTGCAAGGAGAGTC					
